# Phytochemical compositions and antioxidant activity of green and purple basils altered by light intensity and harvesting time

**DOI:** 10.1016/j.heliyon.2024.e30931

**Published:** 2024-05-09

**Authors:** Paria Eskandarzade, Mahboobeh Zare Mehrjerdi, Nazim S. Gruda, Sasan Aliniaeifard

**Affiliations:** a Department of Horticulture, College of Aburaihan, University of Tehran, Tehran, Iran; b Department of Horticultural Science, INRES-Institute of Crop Science and Resource Conservation, University of Bonn, 53121, Bonn, Germany; c Controlled Environment Agriculture Center, College of Agriculture and Natural Resources, University of Tehran, Iran

**Keywords:** Antioxidants, Essential oil, *Ocimum basilicum*, Total flavonoid, Total phenol

## Abstract

*Ocimum basilicum* L. is one of the most important medicinal and vegetable crops due to its essential oil, pleasant aroma and taste. In this study, we evaluated the impact of different light intensities, including 100 %, 50 %, and 30 % of natural sunlight, on the growth, phytochemical compositions, and antioxidant activity of green and purple basil cultivars at two different harvest times: early morning and noon. The height of the plant, number of leaves per plant, length of the petiole, diameter of the stem, and fresh and dry weight of the shoot were all reduced by decreasing light intensity in both basil cultivars. When the plants of both cultivars were grown under full light intensity and were sampled at noon, they showed the highest phenolic and flavonoid contents. The highest antioxidant activity was detected in purple basil cultivars grown under 30 and 50 % of sunlight in both harvests. The green basil cultivar showed the highest antioxidant activity when exposed to 30 % sunlight and harvested in the early morning. The highest essential oil content and yield in both basil cultivars were obtained under full sunlight in the early morning harvests. In summary, light intensity and harvest time influence the phytochemical yield, composition, and growth of two studied basil cultivars. Optimal results, particularly for medicinal purposes, were achieved by morning harvesting to maximize the essential oil yield of basils.

## 1 Introduction

Plants produce chemical compounds mainly to protect themselves against insects, fungi, bacteria, and herbivores. Some of these phytochemicals have therapeutic properties [1]. Basil (*Ocimum basilicum* L.) is an important medicinal and aromatic plant, used as a vegetable, belonging to the mint family (*Lamiaceae*). It is an annual plant that grows in different parts of the world [2]. Basil essential oils are responsible for the fragrance as well as for biological activities [3]. The antibacterial, antioxidant, antifungal, and anti-inflammatory activities of basil species promote consumer health benefits [4]. Various scents from different basil cultivars result from variations in their essential oils. It has been shown that basil essential oil constituents are usually divided into three groups: phenylpropanoids, monoterpenes and sesquiterpene [5]. Phenylpropanoids are the major components of basil essential oils, which make up 90 % of the essential oil. They mainly include eugenol, chavicol, methyl eugenol, methyl chavicol, myristicin, methyl cinnamate, and elemicin, which are responsible for giving medicinal properties [6]. The basil extract possesses phenolic compounds and antioxidant activity [7]. Caffeic acid and rosmarinic acid are the basil essential oil's main phenolic compounds [8].

Production of plants in controlled environment attracted so much of attention due to more controllability over environmental cues [9,10]. Genetic and environmental factors affect plant growth and the content and composition of biologically active compounds. Light, as one of the most critical environmental factors, provides the energy for photosynthesis. Photosynthesis determines plant growth and yield [11]. It regulates plant developmental processes, including seed germination, seedling establishment, transition to flowering stage, and adaptation to stressful conditions [12]. The biosynthesis of secondary metabolites in medicinal and aromatic plants can be strongly influenced by various factors related to light, such as light intensity, quality, and duration [13]. Recently, the production of herbs and medicinal plants in greenhouse or other protected cultivation attracted attention. However, optimization of lighting environments in those types of cultivation is challenging and needs more investigation. For instance, low light intensity decreases the growth rate and biomass production, increasing greenhouse production costs. On the other hand, high light intensity increases greenhouse temperatures during the spring and summer, leading to crop damage [14,15]. Greenhouse shading is standard during hot seasons in the Mediterranean and Middle East. Applying shade nets outside or inside greenhouses is a usual practice in many countries to facilitate control over the temperature inside greenhouses. Therefore, net shades are used to control the temperature. However, shading negatively impacts plant growth and the production of bioactive compounds. For instance, decreasing light intensity in the growth environment caused a reduction in the biomass in *Salvia officinalis* L. [16], essential oil productivity in *Ocimum gratissimum* [17] and phenol production and antioxidant activity in *Labisia pumila* and *Brassica campestris* ssp [18,19]. On the other hand, there is not a clear understanding of the impact of reducing light intensity using shading nets when a cooling system already regulates the greenhouse temperature.

Harvesting time is another critical factor influencing the type and composition of secondary metabolites [20,21]. Temperature and light intensity change throughout the day, with maximum occurring at midday. Diurnal changes in environmental cues would be reflected in the concentration of secondary metabolites. For instance, it has been shown that secondary metabolites of medicinal plants, including the *Lamiaceae* family, vary throughout the day [[22], [23], [24], [25], [26]]. The optimal time to harvest lemon balm and thyme for the highest essential oil is early morning [27]. The highest amount of thymol (the principal constituent of the thyme essential oil) has been detected between 6 and 11 a.m [28]. Padalia et al. (2016) demonstrated that harvesting time affects vital oil yield and composition of four *Ocimum* species. Their study on *Ocimum basilicum* growing in the foothills obtained the highest essential oil yield at noon [23].

Light quantity (e.g. light intensity) can significantly influence plants' morphological characteristics and secondary metabolite production, thereby influencing the quantity and quality of the plant's chemical compositions. We hypothesise that environmental factors such as temperature, humidity, and light intensity can vary significantly over a day. These factors can affect the plant's metabolic activity, synthesis and accumulation of secondary metabolites, such as antioxidant activity and essential oils. Therefore, harvesting plants at different times of the day may result in variations in these compounds, ultimately impacting the quality of the harvested produce. Another objective was to elucidate the response of plants to a controlled temperature range while simultaneously imposing limitations on light intensity through shade nets. Therefore, this study investigates the effects of harvesting time and light limitation through shade nets on the yield and quality of green and purple basil. By examining the total phenolic and flavonoid content, antioxidant activity, and essential oil composition and yield, we aimed to identify the optimal combinations of these factors to obtain the maximum phytochemical quantity and quality.

## 2 Materials and methods

### 2.1 Plant materials

The seeds of green and purple cultivars of basil, respectively ‘Mobarake’ and ‘Ardestan’ from Pakan Bazr, Company (Isfahan, Iran), were sown on a plug tray filled with a mixture of coir-pith and perlite in a ratio of 3:1Seedlings were irrigated during the vegetation with half-strength of Hoagland solution. After the development of the first six leaves, basil seedlings were planted in pots (15 cm depth and 10 cm diameter) containing soil, sand and peat in a 1:1:1 ratio and irrigated three times per week.

### 2.2 Growing conditions

Plants were grown under natural light in a greenhouse compartment during the spring season with day/night temperatures of 24/22 °C. Three weeks after transplanting, plants of each cultivar were subjected to 100 %, 50 % and 30 % natural sunlight. Reduction of the sun was obtained by shading with green plastic nets. Table 1 shows the average photosynthetic photon flux density (PPFD) of different treatments at midnight in the vegetative growth phase of plants, which was measured using a light meter (Sekonic C-7000, Japan). Daily Light Integral (DLI) under control conditions were 27 mol m^−2^ s^−1^. Dou et al. (2018) recommended 12.9 mol m^−2^ d^−1^ to produce basil plants successfully [29]. Since we have measured the PPFD during different times of the day and see, on average, 50 % and 60 % reduction in PPFD by 50 % and 30 % shade treatments, respectively, we presented the values of DLI based on the indicated calculation in Table 1. Two weeks after light treatments, plants were harvested at two different times of the day, in the early morning at 4 a.m. and noon at 12 a.m.

**Table 1:** Photosynthetic photon flux density and daily light integral under 100 %, 50 % and 30 % of natural sunlight in a greenhouse compartment at midnight.

Light treatment	100 % of sunlight	50 % of sunlight	30 % of sunlight
Photosynthetic photon flux density (μmol m^−2^ s^−1^)	1230	615	369
Daily light integral (mol m^−2^ d^−1^)	27	13.5	10.8

### 2.3 Plant growth measurements

Growth parameters of the two basil cultivars, including plant height, number of leaves per plant, petiole length, stem diameter and fresh and dry weights of shoot, were recorded two weeks after the start of light treatments. Shoot dry weights were measured after oven-drying at 80 ^°^C for 48 h.

### 2.4 Preparation of methanolic extracts

One gram of the powder of each plant sample (Leaf No. 2–4 from the top), which was kept at −70 °C, was extracted with 10 mL of 80 % methanol. After 24 h maceration at room temperature (24 ^°^C), following slow shaking, the samples were centrifuged (15 min at 4000 g), and the supernatants were used for further studies.

### 2.5 Determination of total phenolic content

The total phenolic content was evaluated by the Folin-Ciocalteu reagent method [30]. 300 μL of the methanolic extracts were mixed with 4700 μL of distilled water and 200 μL of Folin-Ciocalteau solution. After 2 min, 2 mL of Na_2_CO_3_ (7.5 % w/v) was added, and the samples were placed in the dark for 15 min at 45 °C. The absorbance of all samples was measured at 765 nm using a UV–Vis spectrophotometer (Lambda 25- UV/Vis spectrometer, USA). The total phenolic content was calculated as mg of gallic acid equivalents (GAE) per g fresh weight using a gallic acid calibration curve [30].

### 2.6 Determination of total flavonoid content

The total flavonoid content was determined using the aluminium chloride colourimetry method described by Hosseini et al. [30]. 800 μL of the methanolic extract was mixed with 160 μL of 10 % AlCl_3_, 160 μL of 1.0 M potassium acetate, and 1880 μL of distilled water. The reaction mixture was left to incubate in the dark at room temperature for 40 min. After that, its absorbance was measured at 415 nm against blank using a UV–Vis spectrophotometer. Quercetin was used to develop a standard calibration curve, and the TFC was expressed as mg of quercetin equivalents (QE) on a fresh weight basis.

### 2.7 Antioxidant activity

The radical scavenging activity was assayed using the DPPH method [30]. The methanolic extract (100 μL) was diluted with distilled water (900 μL). Then, 1 mL of 0.1 mM DPPH solution was added, and the samples were placed in the dark for 30 min at 20 °C. The absorbance of the resulting solution was detected at 515 nm using a UV–Vis spectrophotometer. The following equation calculated DPPH-scavenging activity (%):

DPPH-scavenging activity (%) = [(Absorbance of control - Absorbance of sample)/Absorbance of control] × 100.

### 2.8 Isolation of essential oil

The essential oil of plants was obtained using a Clevenger apparatus and hydrodistillation method. Fifty grams of plant material and 500 mL distilled water were added into 1 L flask and distilled for 4 h. The essential oil and the water mixture were finally separated by decantation. Due to the relatively low amount of essential oil in plants grown under 30 % sunlight, critical oil analysis was done only for other treatments.

### 2.9 GC/MS analysis

The essential oils' gas chromatography/mass spectrometry was performed on a Thermoquest gas chromatograph with a flame ionization detector (FID). The equipment has a DB-5 column (30 m × 0.25 mm × 0.25 μm), and the injector temperature was kept at 250 °C. The carrier gas (helium) flow rate was 1.1 mL/min. The oven temperature program was 60–250 °C at the rate of 5 °C min^−1^. The quadrupole mass spectrometer was scanned over 40–460 amu with an ionizing voltage of 70 eV, and the ion chamber temperature was 200 °C. The identification of components was based on comparisons of their mass spectral and retention index with those of the internal reference mass spectra library. The percentage composition of compounds in the essential oil was calculated by area normalization of the GC/FID signal without corrections.

### 2.10 Statistical analysis

We arranged the experiment factorially, employing a completely randomized design. There were ten replications for growth evaluations, while for phytochemical assessment, there were three replications. Statistical analysis was performed using SAS software (SAS Institute Inc.; Cary, N.C.). Data were subjected to two-way analysis of variance (ANOVA) and expressed as mean ± standard error (SE). The mean values were compared using the Duncan test at P < 0.05. Correlations were tested using the Pearson correlation coefficient using SPSS software (SPSS Inc., Chicago, USA).

## 3 Results

### 3.1 Plant growth

The growth of both basil cultivars was negatively affected by a decrease in natural light intensity. Both cultivars obtained the highest number of leaves per plant, plant height, petiole length, stem diameter, and shoot fresh and dry weights under full sunlight. The purple cultivar of basil plants exhibited higher growth traits than the green one, except for stem diameter (Table 2).

**Table 2:** Effect of light intensity on growth parameters of green and purple basil cultivars as influenced by different light intensities (100: 100 % sunlight, 50: 50 % of sunlight and 30: 30 % of sunlight).

Growth parameter	Treatment					
	Green cultivar			Purple cultivar		
	100 % of sunlight	50 % of sunlight	30 % of sunlight	100 % of sunlight	50 % of sunlight	30 % of sunlight
Plant height (cm)	33.9 ± 0.6 c	30.2 ± 0.65 d	28.83 ± 0.6 d	44.45 ± 0.5 a	41.1 ± 0.32 b	34.66 ± 0.51 c
Number of leaves per plant	39.16 ± 0.5 b	30 ± 0.78 c	30.83 ± 0.9 c	50.28 ± 0.6 a	49.66 ± 0.4 a	40.83 ± 0.6 b
Petiole length (cm)	1.31 ± 0.06 b	1.28 ± 0.03 b	1.1 ± 0.03 c	2.24 ± 0.08 a	2.23 ± 0.06 a	1.42 ± 0.7 b
Stem diameter (mm)	3.04 ± 0.09 a	2.56 ± 0.04 b	2.4 ± 0.11 b	2.91 ± 0.08 a	2.9 ± 0.07 a	2.48 ± 0.07 c
Shoot fresh weight	9.03 ± 0.65 b	6.91 ± 0.38 c	6.95 ± 0.34 c	11.39 ± 0.0 a	7.7 ± 0.12 bc	7.07 ± 0.78 c
Shoot dry weight	1.39 ± 0.11 b	0.94 ± 0.02 c	0.86 ± 0.03 c	1.79 ± 0.01 a	1.35 ± 0.04 b	0.97 ± 0.09 c

Means followed by the same letter in the same row do not differ significantly and by the different letter in the same row differ significantly. Values within rows followed by the same letter do not differ statistically (Duncan test, P < 0.05).

### 3.2 Total phenolic content

**Fig. 1:**
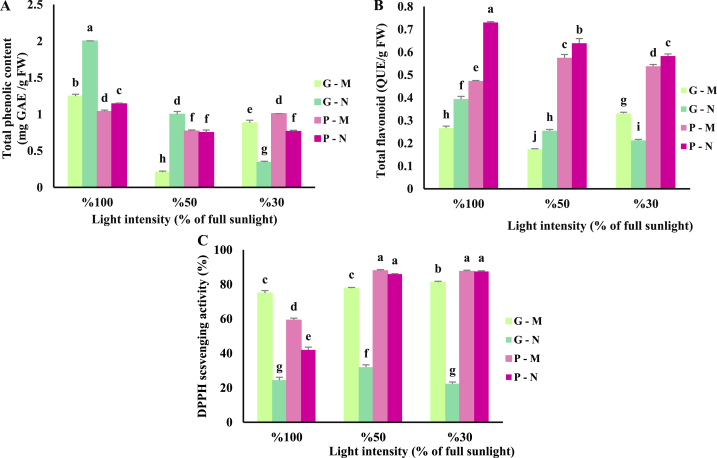
Total phenolic content (A), total flavonoid content (B), and total antioxidant activity (C) of green and purple basil cultivars grown under different light intensities (100: 100 % sunlight, 50: 50 % of sunlight and 30: 30 % of sunlight) and harvested at two times of a day (G–M: Green basil harvested in the early morning, G–N: Green basil harvested at noon, P–M: Purple basil harvested in the early morning and P–N: Purple basil harvested at noon). Various letters (a–j) indicate a notable distinction between treatments (p < 0.05). Bars are means of 3 replications ± SE.

Different light intensities and harvesting times significantly influenced the content of total phenols in both basil cultivars. The green cultivar grown under 100 % sunlight at noon had the highest entire phenolic content of 2 mg GAE/g FW, while the same cultivar had the lowest amount of 0.2 mg GAE/g FW when grown under 50 % sunlight and was harvested in the early morning. The purple cultivar had the highest phenol content of 1.14 mg GAE/g FW when exposed to 100 % sunlight and harvested at noon. Generally, a decrease in light intensity led to a decline in the total phenolic content of both cultivars (Fig. 1 A).

### 3.3 Total flavonoid content

The purple cultivar had the highest flavonoids (0.72 QUE/g FW) when exposed to 100 % sunlight at noon. The total flavonoid content in the purple cultivar was higher than its content in the green cultivar. Thus, the green cultivar had the lowest content of flavonoids (0.17 QUE/g FW) when exposed to 50 % sunlight in the early morning. In general, plants of both cultivars that grew under full sunlight at noon had more flavonoid contents than those grown at lower light intensities in the early morning – a reduction in the light intensity resulted in a decrease in the level of flavonoids. (Fig. 1 B).

### 3.4 Antioxidant activity

The highest antioxidant activity was observed in the purple cultivar grown under 50 and 30 % of sunlight in both early morning and noon harvest times, and the lowest amount was found in the green cultivar under full sunlight and 30 % of full sunlight at noon. In most treatments, the antioxidant capacity at noon was lower than in the early morning (Fig. 1C).

### 3.5 Qualitative and semi-quantitative composition of essential oils

The essential oil percentage and composition were affected by basil cultivar, light intensity and harvesting time. Shading reduced the essential oils' yield and changed the oil composition from the aerial parts of both basil cultivars. The highest essential oil content (1.15 %) and yield (16 mg per plant) were obtained from the green cultivar grown under full sunlight and harvested in the morning. Their lowest amounts belonged to the purple cultivar under 50 % of sunlight at noon (Table 3 and Figure S1). In both cultivars, the percentage and yield of essential oil decreased under reduced light intensities. Higher percentages and yields of essential oil were detected in the morning than at noon. Because of the comparatively low essential oil content in plants cultivated under 30 % sunlight, essential oil analysis was exclusively conducted for the other treatments.

**Table 3:** Chemical composition of essential oils isolated from green and purple basil cultivars grown under different light intensities at additional harvesting time.

RI^a^	RI^b^	Compounds	Compound group	Treatment							
				Green cultivar				Purple cultivar			
				100 % of sunlight		50 % of sunlight		100 % of sunlight		50 % of sunlight	
				M	N	M	N	M	N	M	N
980	981	1-Octen-3-ol (Octenol)	others	0.32	–	0.45	–	–	–	–	0.46
986	981	6-Methyl-5-hepten-2-one (Sulcatone)	others	0.32	–	1.57	–	–	–	–	1.58
995	988	Myrcene	MH	–	2.99	–	–	3.17	–	1.21	–
1032	1024	Limonene	MH	–	3.79	–	0.86	3.47	0.44	–	–
1036	1026	1.8 Cineole	OM	–	–	–	1.34	–	1.5	0.74	–
1087	1083	Fenchone	OM	–	–	–	–	–	–	0.8	–
1101	1095	Linalool	OM	–	6.51	–	–	0.85	5.99	–	–
1146	1141	Camphor	OM	0.32	–	–	–	–	–	–	–
1165	1160	Isocitral<*Z*->	OM	0.93	4.61	1.17	–	4.53	–	–	1.17
1185	1177	Isocitral<*E*->	others	1.37	–	2.12	–	–	–	–	2.12
1197	1186	α-Terpineol	OM	–	8.35	–	–	11.09	1.92	–	–
1206	1195	*p*-Allylanisole (Methyl chavicol)	PP	11.8	2.88	1.19	41.67	7.88	49.9	60.03	1.19
1246	1235	Neral	OM	22.94	18.06	32.08	–	–	–	–	32.81
1254	1249	Geraniol	OM	–	0.73	–	–	–	1	1.5	–
1276	1264	Geranial	OM	31.11	23.4	44.71	–	–	–	–	44.71
1308	1298	Carvacrol	OM	–	–	–	1.33	3.75	–	1.24	–
1379	1374	α-Copaene	SH	0.26	–	0.3	–	–	–	–	0.31
1384	1379	Geranyl acetate	SH	–	–	–	0.97	1.07	0.36	–	–
1423	1419	*trans*-Caryophyllene	SH	2.92	1.59	4.14	1.39	–	1.17	3.3	4.14
1433	1432	*trans*-α-Bergamotene	SH	0.46	–	0.53	1.74	1.42	0.72	0.42	0.53
1451	1440	*Z-*β-Farnesene	SH	0.34	–	0.22	1.43	1.63	–	–	0.23
1459	1452	α-Humulene	SH	–	0.53	1.19	–	–	–	–	1.2
1484	1484	Germacrene-D	SH	1.42	0.42	1.08	2.42	1.78	0.82	0.5	1.08
1499	1500	Bicylogermacrene	SH	–	–	–	1.32	–	0.57	–	–
1520	1522	δ-Cadinene	SH	–	–	0.39	1.03	1.39	0.33	–	0.39
1541	1531	*E*-α-Bisabolene	SH	1.65	0.86	1.38	–	–	–	0.26	1.39
1589	1582	Caryophyllene Oxide	OS	–	–	2.21	–	–	–	1.14	2.22
1624	1628	1,10-di-*epi*-cubenol (cubenol)	OS	–	–	–	3.13	5.87	–	1	–
1662	1652	α-Cadinol	OS	1.4	1.82	0.67	–	–	1.62	–	0.67
1668	1652	α-Eudesmol	OS	–	–	–	3.97	–	3.41	2.91	–
1843	1845	Hexafarnesyl acetone	others	14.1	12.1	2.4	24.92	31.85	18.32	15.09	2.4
2006		Bis(2-ethoxyethyl)ether	others	7.17	11.28	1.38	11.76	20.19	11.84	8.45	1.39
Essential oil content (%)				1.15	0.56	0.73	0.39	0.87	0.16	0.38	0.09
Essential oil yield (mg per plant)				16	7.8	6.8	3.6	15	2.8	5.1	1.2

RI^a^: Retention indices, RI^b^: Retention indices from the literature [31], MH: Monoterpene Hydrocarbons, OM: Oxygenated Monoterpenes, PP: Phenylpropanoids, SH: Sesquiterpene Hydrocarbons, OS: Oxygenated Sesquiterpenes, M: harvested in the morning, N: harvested at noon.

The chemical composition of the essential oils is shown in Table 3. Thirty-two components were detected. The major components of essential oil from the green basil grown under 100 and 50 % of sunlight were geranial, neral and hexafarnesyl acetone, and geranial, neral and *trans*-caryophyllene, respectively in the early morning and hexafarnesyl acetone, geranial and neral, and methyl chavicol, hexafarnesyl acetone and bis (2-ethoxyethyl) ether, respectively at noon. From highest to lowest concentrations, the main compositions of essential oil of the purple basil grown under 100 % and 50 % of sunlight were hexafarnesyl acetone, bis (2-ethoxyethyl) ether and α-terpineol, and methyl chavicol, hexafarnesyl acetone and bis (2-ethoxyethyl) ether in the early morning and methyl chavicol, hexafarnesyl acetone and bis (2-ethoxyethyl) ether, and geranial, neral and *trans*-caryophyllene, respectively at noon. In green basil, limonene was not found in plants harvested in the morning, and its percentage in the essential oil was higher under full sunlight conditions compared to its percentage under 50 % of sunlight at noon. In the purple cultivar, limonene was obtained only under full sunlight both in the morning and at noon. Linalool in the essential oil of the green cultivar was detected only under 100 % sunlight at noon, and in the purple cultivar, it was found under full sunlight in both the early morning and at noon. Still, at noon, its percentage was higher than in the morning.

There was a strong correlation between some of the essential oil compounds of basil and harvesting time (Table 4). For example, a positive correlation was observed between the content of 1.8 cineole and the content of linalool and methyl chavicol. Geranial was shown to have a clear positive correlation with neral. There was also a negative correlation between methyl chavicol and neral and geranial. Positive or negative correlations between some volatile constituents in basil plants indicate predictable relationships among compounds in response to light intensities and harvesting time.

**Table 4:** Correlations between some essential oil compositions in basil plants grown under different light intensities and harvested at other times.

Compound	Myrcene	Limonene	Linalool	Isocitral<E>	α-Terpineol	Methyl chavicol	Neral	Geranial	trans-Caryophyllene	Hexafarnesyl acetone
Limonene	0.91**	1			0.95**				−0.71*	0.71*
1.8 Cineole			0.95**			0.86**				
Isocitral<Z->	0.88**	0.91**			0.92**					
Isocitral<E−>				1			0.91**	0.92**	0.80*	−0.81*
α-Terpineol	0.92**	0.95**			1				−0.72*	0.75*
Neral				0.91**		−0.76*	1		0.74*	−0.77*
Geranial				0.92**		−0.75*	1**	1	0.75*	−0.78*
trans-Caryophyllene		−0.71*		0.80*	−0.72*		0.74*	0.75*	1	−0.94**
Bis(2-ethoxyethyl)ether		0.71*		−0.83**	0.75*		−0.80*	−0.80*	−0.96**	0.99**

Notes: *Correlation is significant at the 0.05 level; **correlation is significant at the 0.01 level.

## 4 Discussion

Basil, abundant in diverse compounds and possessing antioxidant properties, can serve as an extract in vegetable oils, substituting synthetic antioxidants. Moreover, due to its potent antioxidant, diuretic, and antimicrobial properties, it has extensive application as a flavoring agent in diverse foods, oral products, and dental care [7]. As analytical techniques have evolved over the years, the number of identified compounds has increased significantly, with over 140 compounds now known to exist in basil oil. Monoterpenes and phenylpropanoids emerge as the predominant groups present in basil essential oils [8]. Since the levels of phytochemical compounds depends on the environmental cues and the microclimate surrounding the crops, proper managing of environmental cues and harvest can lead to higher levels of phytochemicals in the herbs and medicinal plants.

Light is one of the most critical factors for the plant growth and production of secondary metabolites. Each plant species needs a certain light intensity level for optimal photosynthesis and average growth [32]. Larsen et al. (2020) determine the optimal Daily Light Integral (DLI) for sweet basil (Rosie, Emily and Dolly) production. In the vertical farm, it was suggested to start growing the plants at a PPFD of 150 μmol m^−2^ s^−1^ and increase it to 300 μmol m^−2^ s^−1^ (DLI 19.4 mol m^−2^ d^−1^) towards the end of production [33]. Considering the energy-saving issues, Dou et al. (2018) recommended 12.9 mol m-2 d-1 for the indoor production of basil (Johnny's Selected Seeds, Winslow, ME) [29]. In another study, four light treatments were used, including whole light, 50, and 70 percent of full light. In the treatment with the most shade, plants were shorter, lighter, had smaller leaf surfaces, and fewer branches. Moreover, in the treatment with the most shade, the lowest amount of essential oil was also present [2].

Shading is a widely used technique in horticulture, primarily aimed at controlling temperatures during seasons when they exceed the ideal threshold for plant growth. It is also used to decrease the amount of light exposure for plants that thrive in shaded environments. Shading practice always reduces the quantity and, in many cases, the quality of light reaching the plant surface. In those cases, it reduces the plant's growth depending on the shade level [34].

In the present study, shaded plants were shorter than the complete sunlight-grown plants. Decreased light intensity in the study reduced plant growth; fewer internodes resulted in shorter plants. In a study conducted by Chang et al. (2008), the height of basil plants (Genovese) decreased by reducing the light intensity [2], which was consistent with the results of our study. Plants exposed to low light levels increase their internode lengths to accelerate reaching enough light levels. This response, termed “shade avoidance response”, is an architectural modification in plant structure, and those plants invest more biomass to their internodes rather than to the other parts. Similar to the present study's findings, Poorter et al. (2019) reported that a decrease in internode length due to low DLI exposure is likely due to problems with a deteriorating C-budget [35]. Basil plants cultivated under optimal photosynthetic conditions in our research, i.e., total sunlight exposure, exhibited a significant increase in stature and leaf biomass compared to those grown under shaded conditions. This can be attributed to the abundance of light energy available for photosynthesis, which stimulated the production of internodes, resulting in the observed growth differences. According to Claussen (1996), increasing plant growth in response to increased light radiation is a form of plant adaptation that causes more water and mineral uptake, allowing plants to perform higher levels of photosynthesis and higher transpiration rate [36]. Therefore, the generation of shorter plants under lower DLI can be related to the decrease in internodes.

Leaf developmental rate is one of the most important indicators of plant development, which directly affects the rate of photosynthesis [37]. Our results showed that the highest number of leaves was found under 100 % sunlight in both basil cultivars, and their number reduced under 50 and 30 % sunlight.

Also, the highest dry weight of the plant was obtained under full sunlight intensity in our study. The growth of basil can be adversely affected by reduced light levels that arise from shading practices. This is because low light intensity serves as a limiting factor for its growth, as noted by Fernandes et al. (2013) [17].

Many factors, including temperature, light intensity, harvesting time, and plant genetics, affect the type and chemical composition of plant secondary metabolites [38]. The amount of phenolic compounds and flavonoids produced by plant species can be affected by the intensity of light they receive [39]. One way to prevent sun damage to plants is to increase the level of phenolic compounds in the leaves because phenol acts as an optical filter and prevents damage to the plant [40]. Therefore, it is *unsurprising that the basil plants grown under full sunlight had a higher phenolic content than those* grown under low light intensities. In 100 % of sunlight, the total phenolic content at noon was more significant than their content in the early morning. Still, it was the opposite in the plants exposed to 30 % sunlight, and the highest total phenolic content was detected in samples collected in the early morning. The previous study reported an increase in the phenolic content of *Premna serratifolia* L. followed by an increase in the temperature at noon [41]. Furthermore, an increase in total flavonoid contents has been demonstrated in various medicinal and aromatic plants by exposure to high light intensity [42]. It has been reported that light plays a critical role in flavonoid biosynthesis, and these compounds may be involved in the adaptation of plants to extreme light conditions [43]. In this study, flavonoid contents at noon were increased as the light intensity increased, similar to the results obtained in *Labisia pumila* [18]. In all cases, the purple basil cultivar had higher flavonoid levels than the green one. The superiority of the purple basil over the green basil in flavonoid contents is also mentioned by Tenore et al. [44].

The antioxidant activity of both basil cultivars was found to be elevated by decreasing light intensity. This increase in antioxidant activity may be due to the accumulation of ROS in the plant tissues due to low light stress [45]. In the green basil cultivar, the plants harvested in the early morning had more antioxidants than at the noon. In a study on *Premna serratifolia*, similar results were obtained that correlated with temperature changes, so it was stated that there is an inverse relationship between the antioxidant levels and the temperature; if the temperature elevates (at noon), the antioxidant levels decrease [41]. In another study conducted on basil (cv. Genovese) plants, it was also found that the reduction in light intensity by shading increased the antioxidant activity [46]. Sweet basil species have notable antioxidant potential and could serve as natural preservatives. The adjustment of light intensity, facilitated through shade nets, emerges as a physiological tool to enhance phytochemical quality and antioxidant activity [47].

Despite an increase in the level of flavonoids, the antioxidant activity of the plants decreased in the noon time in the present study. This can be due to the particular function of flavonoids. Their production increases due to high light stress in plants primarily as a protective response to oxidative damage caused by excessive light exposure. In response to high light stress and the associated oxidative damage, plants activate defence mechanisms to counteract the detrimental effects. One such defense mechanism is the upregulation of flavonoid biosynthesis. Under high light stress conditions, plants increase the production of enzymes involved in flavonoid biosynthesis, such as phenylalanine ammonia-lyase (PAL) and chalcone synthase (CHS) [48].

Close correlations have been reported between the light characteristics and the production of essential oils in plants [49]. The results of the present study confirmed a decrease in essential oil production by reducing the light intensity levels. The accumulation of essential oils in plants directly or indirectly depends on the intercepted light level. Promotion of growth can increase the yield of essential oil [50]. As light intensity decreases, the rate of photosynthesis and plant growth decreases, which reduces the percentage and yield of essential oil [2]. It has been shown that harvesting at different times during the day affects the amount of essential oil and its compounds [28]. This could be due to temperature or light level changes during the day. Changes in temperature can affect plant growth and productivity by stimulating physiological processes and the biosynthesis of plant secondary metabolites triggers in the presence of temperature stress [51]. In the present experiment, there were no significantly different temperatures under the shadings due to enough ventilation in the greenhouse compartment. The difference in the type and percentage of basil essential oil compounds can be due to genetic factors or the nutritional status of the plant as well. Methyl chavicol, linalool, methyl cinnamate, methyl eugenol, eugenol and geraniol are the main constituents of essential oils of most basil cultivars [52]. It has been reported that the composition of the essential oil can be significantly affected by light intensity [2]. The synthesis of these aromatic compounds is closely related to the primary metabolism of the plants, which results in more photoassimilate production due to the induction of photosynthesis, leading to more production of secondary metabolites [53]. For instance, phenylalanine is made by a group of enzymes, such as sucrose synthases and glucose-6-phosphate dehydrogenases. An increase in the production of sucrose in the plant facilitates the production of secondary metabolites derived from it [54]. In a study on the basil (Genovese) by Chang et al. (2008), plants that were exposed to more light had higher levels of linalool [2], which was consistent with the results of this study. Many of the toxic effects of basil essential oil have been attributed to methyl chavicol [55]. The percentage of this compound under full sunlight was higher in the green and purple basils in the early morning and at noon, respectively. Therefore, to improve the quality and quantity of the basil essential oil, it is important to pay attention to light intensity and harvesting time.

## 5 Conclusions

Light, as the driving force of photosynthesis, affects plant growth, yield, and the production of secondary metabolites. The results of this study showed that, in general, the purple basil cultivar had more biomass than the green cultivar. Decreased light intensity, and as a result, DLI reduced plant growth in both cultivars. It is crucial to acknowledge that shading can significantly decrease basil yield by hindering photosynthesis and diminishing the production of vital primary metabolites. Harvesting time is one of the environmental factors influencing the amount and type of plant secondary metabolites. Antioxidant levels (DPPH free radical inhibition rate) were higher in the green cultivar in the early morning than at noon under all light intensities. However, their levels in the purple cultivar did not significantly differ between morning and noon under different DLIs. Under full sunlight, the phenol and flavonoid contents were higher at noon than in the early morning, which can be due to their protective roles against intense light at noon. Consequently, due to the decrease in the production of primary metabolites and growth, which was followed by a decrease in the percentage of essential oil resulting from reduced light intensity and DLI by shading practices. Therefore, it is recommended to grow basil under full sunlight and harvest it in the morning to achieve the highest amount of essential oil under greenhouse conditions.

## Funding

We would like to thank 10.13039/501100004481University of Tehran for supporting this work.

## Data availability statement

Data will be made available on request.

## CRediT authorship contribution statement

**Paria Eskandarzade:** Writing – original draft, Validation, Methodology, Formal analysis. **Mahboobeh Zare Mehrjerdi:** Visualization, Validation, Supervision, Project administration, Investigation, Formal analysis, Conceptualization. **Nazim S. Gruda:** Writing – review & editing, Validation, Supervision, Investigation, Funding acquisition. **Sasan Aliniaeifard:** Writing – review & editing, Visualization, Validation, Supervision, Project administration, Investigation, Conceptualization.

## Declaration of competing interest

The authors declare that they have no known competing financial interests or personal relationships that could have appeared to influence the work reported in this paper.
