# Supplementary material for: Phytochemical compositions and antioxidant activity of green and purple basils altered by light intensity and harvesting time
Source: Heliyon. 2024 May 9;10(10):e30931. doi: 10.1016/j.heliyon.2024.e30931 (PMC11168194; doi:10.1016/j.heliyon.2024.e30931)
Supplement: Multimedia component 1 [file mmc1.docx]

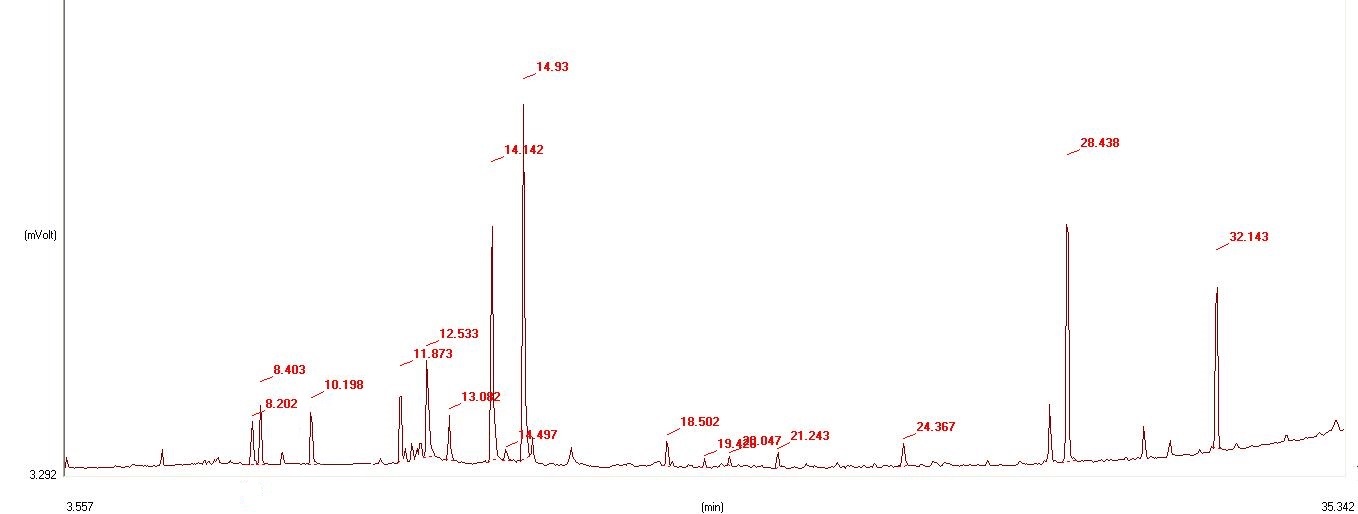


C


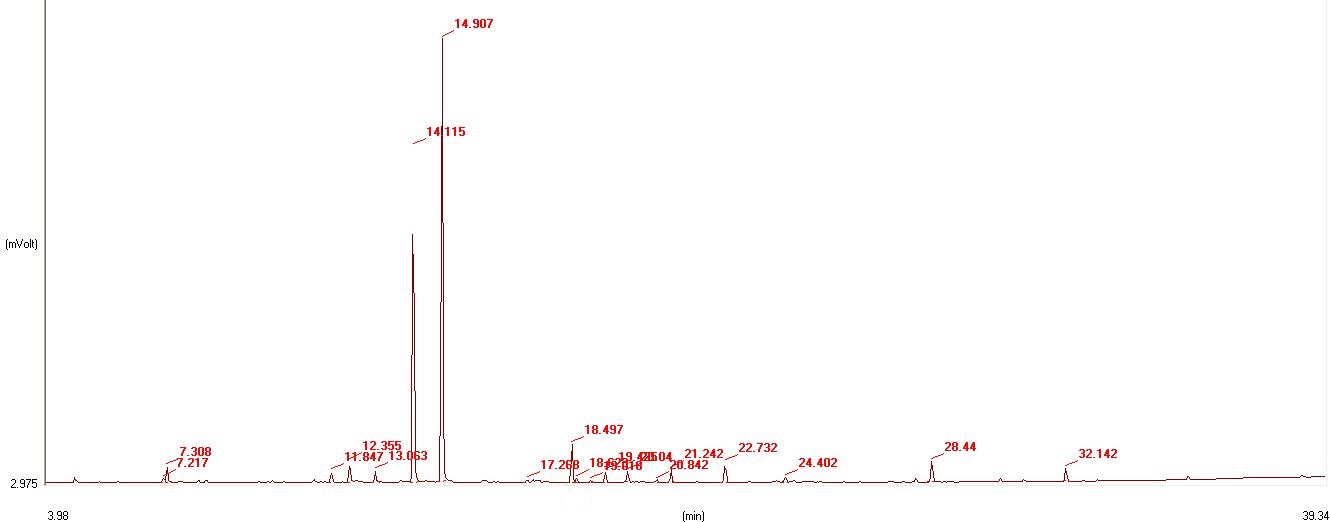


B


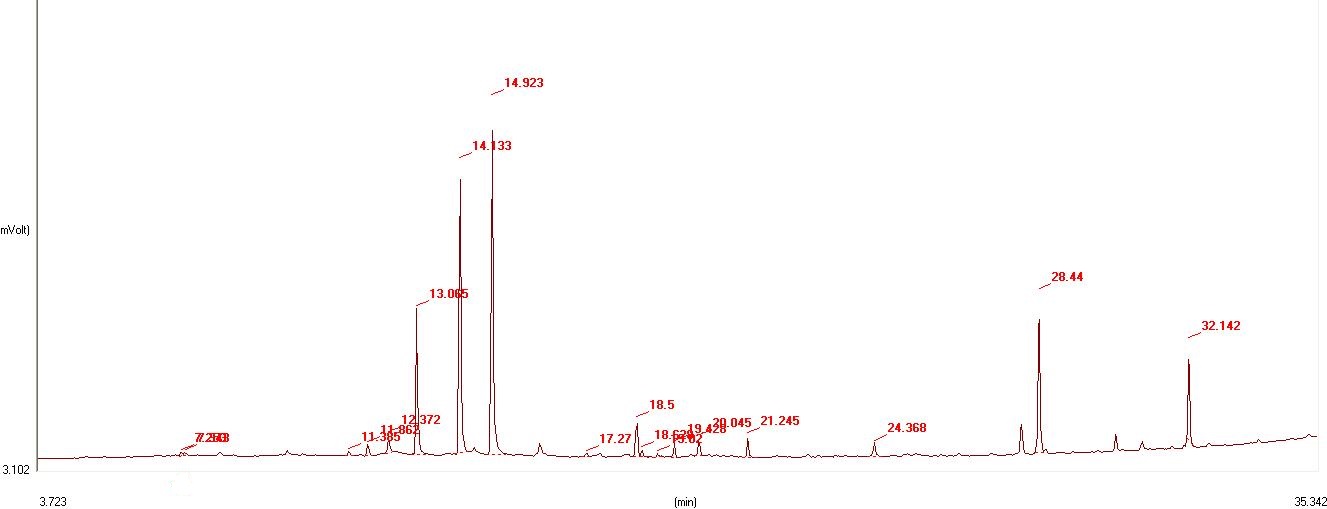


A


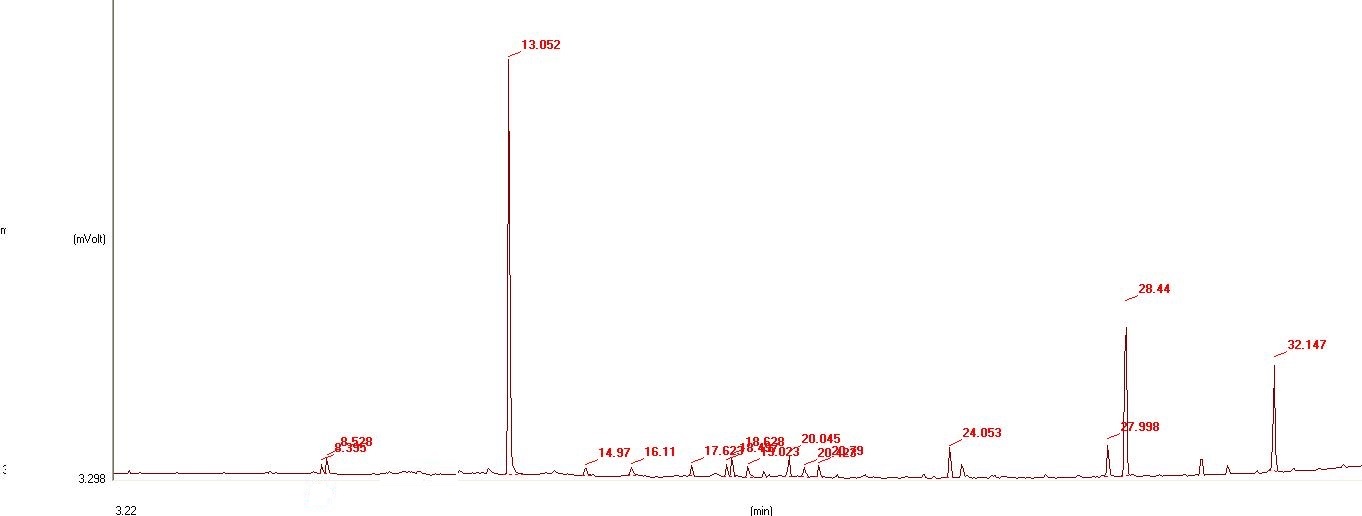


D


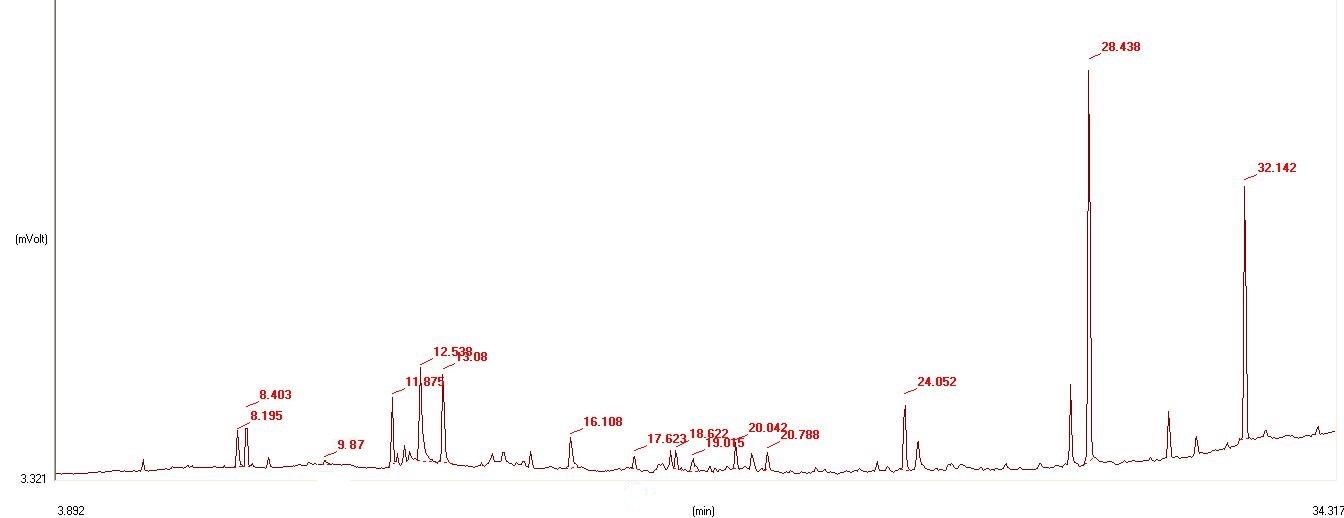


E


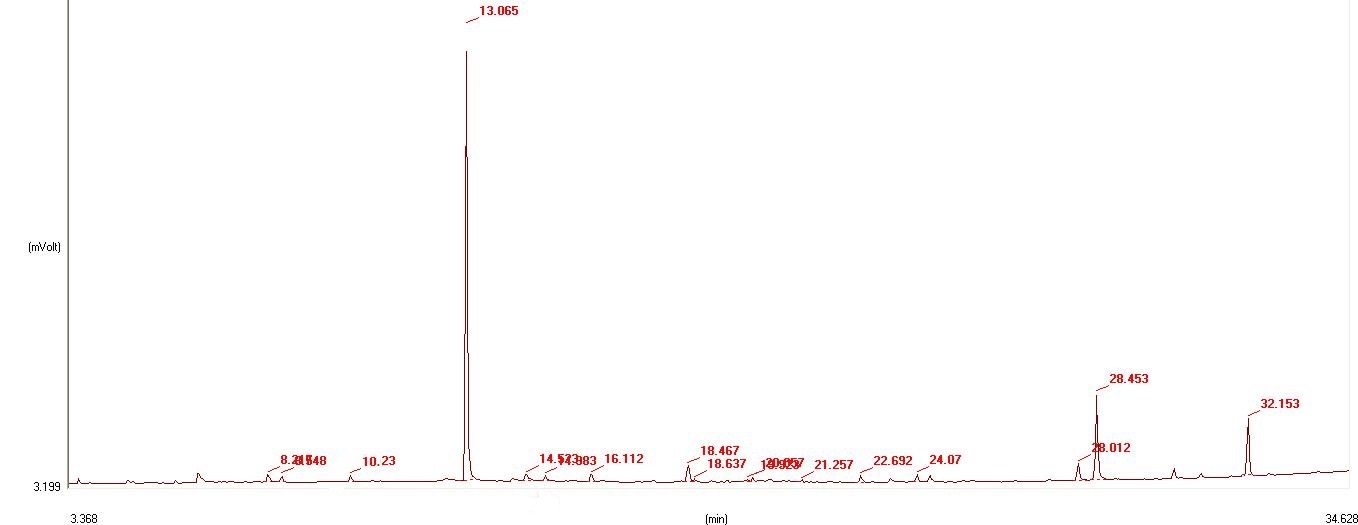


F

H


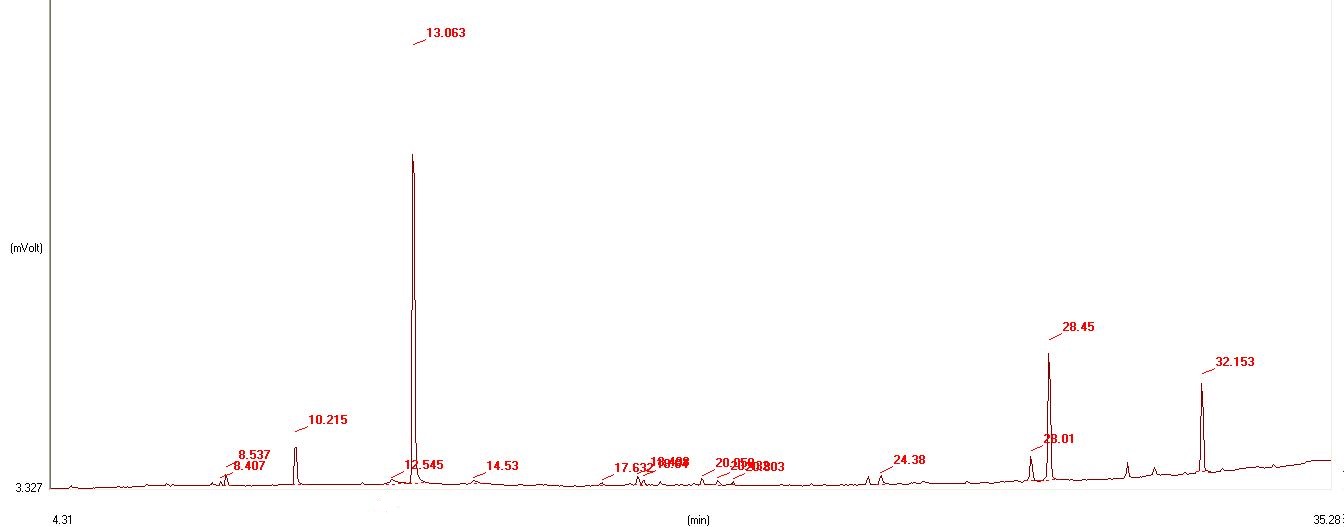


G

Figure. S1. GC/FID chromatograms (A-G) and GC/MS chromatogram (H) of essential oils were examined using gas chromatography/mass spectrometry (GC/MS). Green basil, 100% of sunlight and harvested at the morning (A)., Green basil, 50% of sunlight and harvested at the morning (B)., Green basil, 100% of sunlight and harvested at the noon (C)., Green basil, 50% of sunlight and harvested at the noon (D)., Purple basil, 100% of sunlight and harvested at the morning (E)., Purple basil, 50% of sunlight and harvested at the morning (F)., Purple basil, 100% of sunlight and harvested at the noon (G)., Purple basil, 50% of sunlight and harvested at the noon (H).
